# Quantitative Definition of Low-Health-Interest Populations by Using Regression Trees: A Nationwide Internet Survey in Japan

**DOI:** 10.3390/ijerph21081049

**Published:** 2024-08-09

**Authors:** Yoko Nishizawa, Takuya Yamada, Kumi Sugimoto, Chie Ozawa, Takahiro Tabuchi, Hirono Ishikawa, Yoshiharu Fukuda

**Affiliations:** 1 Teikyo University Graduate School of Public Health, Itabashi-ku, Tokyo 173-8605, Japan; yamada.takuya.ac@teikyo-u.ac.jp (T.Y.); hirono-tky@umin.ac.jp (H.I.); fukuday@med.teikyo-u.ac.jp (Y.F.); 2 Department of Medicine, Tokyo Women’s Medical University Adachi Medical Center, Adachi-ku, Tokyo 123-8558, Japan; 3 Center for Occupational and Environmental Health, Teikyo University, Itabashi-ku, Tokyo 173-8605, Japan; kumi.sugimoto@teikyo-u.ac.jp; 4 Division of Cancer Information Service, National Cancer Center Japan Institute for Cancer Control, Chuo-ku, Tokyo 104-0045, Japan; 5 Cancer Control Center, Osaka International Cancer Institute, Chuo-ku, Osaka 541-8567, Japan; tabuchitak@gmail.com; 6 Division of Epidemiology, School of Public Health, Tohoku University Graduate School of Medicine, Sendai-shi 980-8575, Japan

**Keywords:** health interest, health behavior, regression tree, interest in health scale, receiver operating characteristic curve

## Abstract

Background: Reducing health disparities is a public health issue. Identification of low-health-interest populations is important, but a definition of people with low health interest has not yet been established. We aimed to quantitatively define low-health-interest populations. Methods: A nationwide cross-sectional internet survey was conducted in 2022. We compiled regression tree (RT) analyses with/without adjustment for age, sex, and socioeconomic status with the 12-item Interest in Health Scale (IHS, score range 12–48) as an explanatory variable and the 10 composite health behaviors as a dependent variable. We defined the first IHS branching condition from the root node as a lower-health-interest group and the terminal node with the lowest health behaviors as the lowest-health-interest group. Results: The mean IHS value of 22,263 analyzed participants was 32.1 ± 5.6; it was higher in females and in those who were aged over 45 years, had a high education, a high income, or a spouse. The first branching condition was IHS 31.5, and the terminal node branched at 24.5, before/after adjustment for covariates. Conclusions: We determined the cutoff values of the IHS as <32 for a lower-health-interest group and <25 for the lowest-health-interest group. Using these cutoffs might enable us to reveal the characteristics of low-health-interest populations.

## 1. Introduction

Reducing health disparities is a public health issue. Population approaches, which involve intervening in the entire population regardless of its risk stratification, have the potential to widen health disparities [1,2,3]. In other words, although the population approach promotes health behaviors among people who have a naturally high interest in health [4,5,6], it does not adequately intervene in the “health-uninterest population [7]”, who have more high-risk factors and are reluctant to engage in health behaviors. Various factors are involved in these behaviors, including age, sex, socioeconomic status, and health literacy [8,9,10].

To reduce health disparities, it is important to identify populations that are uninterested in health and to encourage them to switch to health behaviors [11,12]. The transtheoretical model has revealed that people’s health behavior change involves progress through six stages: precontemplation, contemplation, preparation, action, maintenance, and termination [13]. According to this model, people without health behaviors are not just categorized into one group but can be classified into three different categories: precontemplation, contemplation, and preparation stages. Therefore, we suspected that even among people who are not willing to engage in health behaviors, there might be differences in the levels of health interest. Moreover, different types of approaches or strategies are needed to make people change effectively toward health behaviors [14]. For example, for people who are not interested or have no motivation at all to change their health behaviors (precontemplation stage), it is important to make them aware of their health. On the other hand, for people who are willing to change their health behaviors but have not yet changed (contemplation and preparation stages), it might be effective to make them realize that health behavior change is for their own benefit. However, the lack of an established definition to date of people with low health interest makes it difficult to identify them, understand their characteristics, and plan effective interventions directed at them. Recently, a 12-item scale for measuring health interest, namely the Interest in Health Scale (IHS), was developed for Japanese people to quantitatively evaluate their interest in health [15]. Previous studies have revealed that a high score on the IHS is associated with health behaviors, and vice versa [15,16].

A regression tree (RT) is a type of decision tree that uses a continuous variable as a dependent variable. The data are divided into two groups depending on the branching conditions for the variable selected from among the explanatory variables [17,18,19,20]. The RT is also used to build clinical prediction models, and many studies have reported that it performs as well as, or better than, models developed by using traditional statistical methods [21,22,23]. An RT can also be used to build cutoffs for biomarkers or scores [24,25]. It has the advantage of revealing complex interactions between explanatory variables for which it is difficult or impossible to reveal associations by using traditional multivariable techniques [18].

Here, we aimed to determine the cutoffs of the IHS for the low-health-interest population, with a total score of 10 composite health behaviors as an outcome, by using an RT with adjustment for sex, age, and socioeconomic status.

## 2. Materials and Methods

### 2.1. Study Design and Population

We utilized data from the Japan “Society and New Tobacco” Internet Survey (JASTIS) 2022. Details of the protocol of the overall JASTIS study are given by Tabuchi et al. [26]. This cross-sectional study was conducted between 1 and 28 February 2022. A web-based, self-reported-questionnaire survey was distributed by one of the largest Japanese internet research agencies, which has about 2.2 million panelists [27]. From among 43,712 panelists who had participated between 2015 and 2021 in JASTIS or its sister study, JACSIS (Japan “COVID-19 and Society” Internet Survey [28]), 3714 were lost to follow-up. The remaining 39,998 panelists were invited to participate in the 2022 JASTIS questionnaire (follow-up survey). Of these, 11,222 people did not respond. We therefore invited new panelists randomly selected and stratified by age, sex, and prefecture (new survey) until the total number reached 33,000.

**Figure 1:**
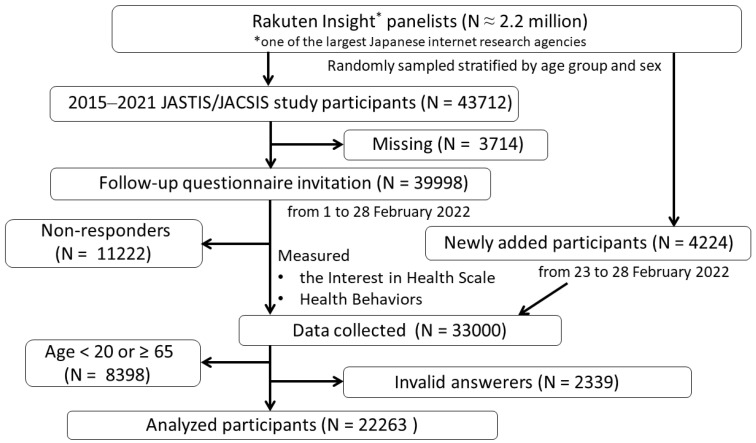
Flowchart of this study. Abbreviations: JACSIS, Japan “COVID-19 and Society” Internet Survey; JASTIS, the Japan “Society and New Tobacco” Internet Survey.

In the analysis, we restricted the analyzed participants to those aged between 20 and 64 years. This was because our outcomes included drinking and smoking behaviors, and both of these are legally allowed from the age of 20 in Japan, and also because health interest is known to be higher in older Japanese people [29]. Moreover, we excluded invalid answers (N = 2339), so that finally the data from 22,263 participants were analyzed. Invalid answerers were defined as follows: (i) those who wrongly answered the question, “Please select the second to last option from the following five choices”; (ii) those who answered “yes” to all of the questions regarding the use of alcohol and nine drugs, including eight illegal drugs; and (iii) those who answered “yes” to all of the questions regarding having nine kinds of chronic illnesses (Figure 1).

### 2.2. Consent and Ethics

General consent from the panelists for the internet survey was obtained from the research agency in advance. In addition, we explained the significance and purpose of the study, and we posted voluntary participation. Participation in the study was taken as consent, but if there were any questions that participants did not wish to answer, they were given the option of “I do not want to answer”, or the option of discontinuing the questionnaire, even in the middle of the survey. The study was conducted in accordance with the guidelines of the Japan Marketing Research Association, and it was approved by the Osaka International Cancer Center Ethics Committee (approval number: 1611079163-2; and approved date: 8 January 2020) and the Teikyo University Ethics Committee (approval number: 22-199; and approved date: 30 March 2023).

### 2.3. Measures

#### 2.3.1. Basic Participant Characteristics

Participants were asked their sex, age, education, occupational status, annual household income, and marital status as background information. Each was defined as follows: Sex was assumed to be based on anatomical structure, and only a binary sex option was presented. Educational background was categorized into five categories based on the last school from which the participant had graduated: (i) junior high school; (ii) high school (private/public/national); (iii) vocational school/college/technical college; (iv) university or higher (private/public/national/graduate school); and (v) others. Occupational status was categorized into five categories: (i) full-time employee (manager/non-manager); (ii) part-time employee (part-time worker/freelance/temporary worker/contract worker/one-off job undertaken through online platforms); (iii) self-employed/employer (self-employed owner/company executive); (iv) non-worker (retired/housewife/househusband/unemployed); and (v) student (students including employed part-time jobs). Annual household income was categorized into six categories: (i) less than 3 million yen; (ii) 3 to less than 5 million yen; (iii) 5 to less than 7 million yen; (iv) 7 to less than 10 million yen; (v) 10 million yen or more; and (vi) I don’t know/I don’t want to answer. Marital status was categorized into four categories based on current status: (i) married (including common-law marriages); (ii) never married; (iii) widowed; and (iv) divorced.

#### 2.3.2. Interest in Health Scale (IHS)

We administered a 12-item IHS to assess the participants’ interest in health [15]. The IHS is composed of three factors, namely health consciousness, motivation, and value. Its validity, reliability, and internal consistency have been confirmed, and its association with health behaviors has been reported in previous studies [15,16]. Each item’s responses were converted into scores from 1 to 4 by using a Likert method, and the total score was used as the IHS score (score range, 12 to 48). Details of the IHS questionnaire can be found in Appendix A, Table A1.

#### 2.3.3. Health Behaviors

There is no single health behavior for which there is absolute consensus regarding its definition as a health behavior. Moreover, because our goal was to identify a higher-risk population that had multiple accumulated unhealthy behaviors, we decided to use a combination of multiple health behaviors as an outcome. As a dependent variable, we asked for responses to questions regarding the following 10 statements about health behaviors, with reference to the past literature [8,30,31,32,33,34,35]: (i) currently non-smoker; (ii) no harmful alcohol use; (iii) sleep for at least 6 h; (iv) not obese; (v) eat breakfast; (vi) have a nutritionally balanced diet; (vii) maintain a regular routine; (viii) brush teeth; (ix) have received a medical checkup within the past year; and (x) have received a dental checkup within the past year. Details of the questionnaire content for each health behavior can be found in Appendix A, Table A2.

Regarding sleep duration, taking into consideration the fact that the target sleep duration for Japanese is 6 to 8 h [36], we decided that a sleep duration of at least 6 h was a healthy behavior. Also, physical activity is considered a health behavior in some studies [8,30,31,33]. However, our study was conducted during the Coronavirus 2019 pandemic, and people’s trends toward physical activity had negatively changed [37,38,39]. Taking this into consideration, we decided not to include physical activity as a health behavior. The response to each of the 10 statements that best reflected each healthy behavior was awarded one point, and the total score was calculated as the health behavior score (score range, 0 to 10).

### 2.4. Statistical Analysis

#### 2.4.1. Basic Participant Characteristics

Continuous variables are reported as means (standard deviation, SD) for normally distributed data or medians (inter-quartile range, IQR) for non-normally distributed data. Discrete variables are expressed as numerals (percentages). Subjects were allocated to groups according to their background characteristics. The differences in IHS scores between the groups were tested by using Welch’s *t*-test for normally distributed data, the Wilcoxon rank sum test for non-normally distributed data, and a one-way analysis of variance if there were three groups or more.

#### 2.4.2. Regression Tree (RT) Analysis

In the RT analysis, the dependent variable was the total score for the 10-item health behaviors (score range [integer], 0 to 10) and the explanatory variable was the IHS score (score range [integer], 12 to 48). We created an RT by using the Gini index [40,41] for the overall group, and then another RT after stratification by sex. We defined the first branching condition from the root node as the cutoff for a lower-health-interest group, and the branching condition to the terminal node with the lowest health behavior score as the cutoff for the lowest-health-interest group. We added a similar RT analysis stratified by sex and by 10-year age groups. Moreover, we conducted an RT analysis with adjustment for age and socioeconomic status (education, occupational status, annual household income, and marital status) by adding these variables as explanatory variables to the RTs. In all models, we did not trim any branches and did not give any conditioning rules to the RTs.

#### 2.4.3. Sensitivity Analysis: Receiver Operating Characteristic (ROC) Curve

As a sensitivity analysis, we added an analysis using an ROC curve with each of the 10 individual health behavior scores (0 or 1 point) as a dependent variable and the IHS score as an explanatory variable. We drew the ROC curve, calculated the area under the curve (AUC), and determined the cutoff value by using the Youden-Index.

#### 2.4.4. Classification: Participant Characteristics Categorized by Determined Cutoff Values

We allocated the participants to groups by using the cutoff values we obtained, and we then calculated the number of people and the proportions of characteristics in each group. In the analyses, no imputation methods were used, and available-case analyses were done. A two-tailed *p*-value of <0.05 was considered to indicate statistical significance. Statistical analyses were performed by using IBM SPSS Statistics version 28.0.1.0 and R software version 4.1.3.

## 3. Results

### 3.1. Basic Participant Characteristics

Table 1 shows the baseline characteristics of the 22,263 participants and a summary of the IHS values for each background characteristic. The mean overall IHS score was 32.1 (SD 5.6); it was significantly higher in females than males (32.7 and 31.6, *p* < 0.001). Those who were aged 45 years or more, had a high level of education, or had a high household income had significantly higher IHS scores, whereas those who were students, unmarried, or divorced had significantly lower scores.

### 3.2. RT Analyses of the Total 10-Item Health Behavior Scores and the IHS

First, when we calculated the health behavior score the responses of those who answered “I don’t know” about their sleeping hours (N = 426) were treated as missing values. The overall median health behavior score was 8 (IQR 7 to 9); the median for males was 7 (IQR 6 to 9); and the median for females was 8 (IQR 7 to 9).

**Figure 2:**
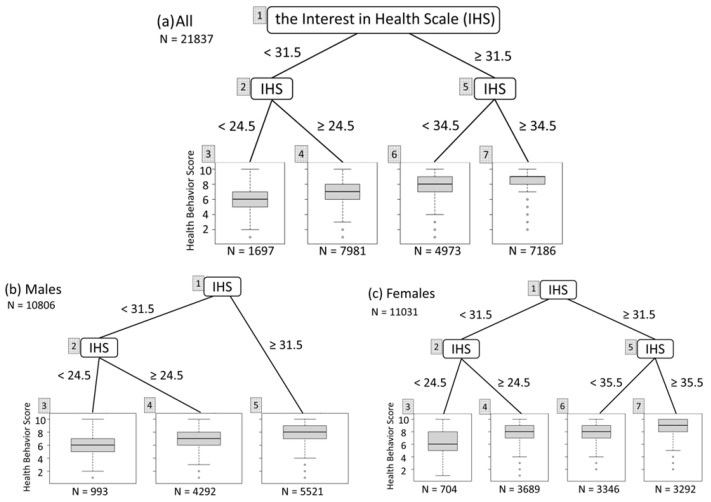
Regression tree analyses for health behaviors and the Interest in Health Scale with/without stratification by sex: (**a**) all participants; (**b**) males; and (**c**) females. Each box plot represents the median, interquartile range, the smallest value greater than the lower quartile minus 1.5 times the inter-quartile range, and the largest value less than the upper quartile plus 1.5 times inter-quartile range of the health behavior score at a terminal node.

Secondly, we plotted the results of the overall and sex-stratified RT analyses of the IHS scores. The overall RT branched at the conditions of IHS < 31.5 from the root node and <24.5 for the terminal node with the lowest health behaviors (Figure 2a). When stratified by sex, it also branched at <31.5 from the root node and <24.5 for the terminal node with the lowest health behaviors, in both males and females (Figure 2b,c). Further stratification by sex and age group revealed that males aged 20–24, 25–34, 35–44, 45–54, and 55–64 years had IHS branching conditions of 31.5/29.5 (first branching condition from the root node/branching condition to the terminal node with the lowest health behaviors), 31.5/24.5, 31.5/27.5, 31.5/25.5, and 30.5/27.5, respectively. Females aged 20–24, 25–34, 35–44, 45–54, and 55–64 years had branching conditions of 31.5/24.5, 29.5/24.5, 29.5/24.5, 30.5/24.5, and 30.5/24.5, respectively.

**Figure 3:**
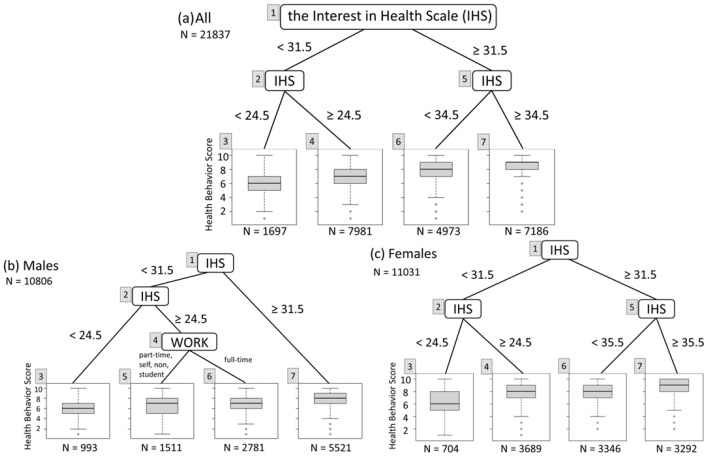
Regression tree analyses for health behaviors and the Interest in Health Scale with/without stratification by sex, after adjustment for age, sex, and socioeconomic status by adding these variables as explanatory variables to the regression trees: (**a**) all; (**b**) males; and (**c**) females. Each boxplot represents the median, inter-quartile range, the smallest value greater than the lower quartile minus 1.5 times the inter-quartile range, and the largest value less than the upper quartile plus 1.5 times inter-quartile range of the health behavior score at a terminal node. Abbreviations: WORK, employment status; full-time, full-time employee; non, non-worker; part-time, part-time employee; and self, self-employed/employer.

Finally, we plotted the results of an RT analysis of the IHS results in which sex, age, and socioeconomic status were adjusted for by adding these variables as explanatory variables to the RTs, either with or without stratification by sex. The overall RT branched at IHS conditions of <31.5 from the root node and <24.5 for the terminal node with the lowest health behaviors (Figure 3a). After stratification by sex, the males’ RT branched at IHS conditions of <31.5 from the root node and <24.5 for the terminal node, as well as by occupational status (full-time employee vs. others). For females, the RT branched at IHS conditions of <31.5 from the root node and <24.5 for the terminal node with the lowest health behaviors, with no branching conditions for socioeconomic status (Figure 3b,c).

Comparison of males and females revealed that males with the highest health behaviors (IHS ≥ 31.5, N = 5221) had a median health behavior score of 8 (IQR 7–9), whereas females had an even higher branching condition at IHS ≥ 35.5 (N = 3292), and their median health behavior score was 9 (IQR 8–10)—higher than that of males (Figure 3b,c).

### 3.3. ROC Curve Analyses of Each Health Behavior and the IHS

Table 2 shows the results of our calculations of AUC by using the ROC curve. The cutoff value for not obese was 30.5, and that for the three items of “currently non-smoker”, “brush teeth”, and “have a dental checkup in the last year” was 32.5. The remaining six items all had a cutoff value of 31.5. The lowest AUC was 0.55, for “no harmful alcohol use”, and the highest was 0.76, for “have a nutritionally balanced diet”.

### 3.4. Participant Characteristics Classified by Cutoff Values

By using the cutoff values that we obtained, participants were classified into three groups: normal- or high-health-interest group (IHS of 32 or above), a lower-health-interest group (IHS of 25 or above and less than 32), and the lowest-health-interest group (IHS of less than 25) (Table 3). There was a tendency for greater percentages of males and those of younger age, lower education, or lower income, who were either never married or divorced, to fall into the lower- and lowest-health-interest groups, rather than the normal- or high-health-interest group.

## 4. Discussion

We used RTs to define the cutoff values of the IHS for defining low-health-interest populations. We determined the cutoff value of the IHS for defining a lower-health-interest group as less than 32 and that for the lowest-health-interest group as less than 25. However, our RT stratified by sex and by 10-year age groups showed that it was possible that these cutoff values could vary among age groups. For females, we found that there was a group that had more interest in health and were engaged in more health behaviors than were males. Moreover, there was a tendency for the percentages of males and those of younger age or lower socioeconomic status to be higher in the lower- and lowest-health-interest groups.

The strength of our study was that we determined the cutoff values for IHS by using RTs with an outcome of multiple health behaviors that were investigated in a cross-sectional design. Although there are several linguistic definitions of the low-health-interest population [2,42,43,44], objective and quantitative evaluations are difficult if a linguistic definition alone is used. In contrast, the use of the IHS enabled us to quantitatively evaluate interest in health. Traditionally, ROC curves have been used as a typical statistical method for creating cutoff values. Bradley [45] suggested that the AUC might be more accurate when a single-number evaluation of machine-learning algorithms was required. Here, we also performed ROC analyses and revealed that the cutoff values for 6 of the 10 factors in the ROC analyses and the cutoff value for a lower-health-interest group as determined by the RT analysis were the same as the integer score cutoff value. On the other hand, the cutoffs of 4 of the 10 health behaviors were different from those obtained in the RT analysis. Specifically, the cutoff value for “not obese” was 30.5, and the cutoff values for “currently non-smoker”, “brush teeth”, and “have received a dental checkup within the past year” was 32.5. We defined obesity as having a body mass index of 25 (kg/m^2^) or more, but as our study participants’ median body mass index was 21.6 (IQR 19.6–24.1); 5893 (17.9%) participants were categorized as obese, but that proportion was lower than that of the Japanese population, which is 33.0% for males and 22.3% for females [46]. It is possible that our study participants included many people who were not obese, even if their health interest was low. Analysis of smoking behaviors revealed that 5812 (26.1%) were current smokers, whereas 7078 (31.8%) were past smokers, meaning that they had successfully quit smoking. It was assumed that past smokers had quit smoking because they cared about their health and might have had increased health interest [47]. In terms of toothbrushing and dental checkups, it has been reported that awareness of oral care is low in Japan, with less than half of people visiting the dentist for annual checkups [48]. This suggests that a higher level of health interest is needed for dental-care-related health behaviors.

A limitation of the ROC analysis was that a dependent variable should be binary [49]. According to the transtheoretical model, there are three stages before people adopt health behavior change (precontemplation, contemplation, and preparation), and different approaches towards each stage are effective in changing these stages into health behaviors [13,14]. Therefore, we suspected people with low health interest of having different levels of health interest, and we aimed to extract the higher-risk population with accumulated unhealthy behaviors. We conducted RT analysis as a main analytical method, by using multiple health behaviors as a composite result, instead of using ROC analysis, and we revealed that there were two cutoff points in the low-health-interest populations. It is possible that people in the lowest-health-interest group were in the transtheoretical model’s precontemplation stage: they were not interested in, or had no motivation at all toward, changing health behaviors; they could be called the “health apathy” population. A disadvantage of RT analysis is overfitting, but a sufficient number of samples were secured for each terminal node, so we consider that our models were appropriate.

Our RT stratified by sex and by 10-year age groups showed that these cutoff values may vary among age groups. For example, for males between the ages of 35 and 64 years, a cutoff value higher than <25 might be necessary to define the lowest-health-interest group. We thought that this result might suggest that males between the ages of 35 and 64 years were not able to implement health behaviors, even though they might have had a certain level of health interest. In fact, it has been shown that males in these age groups may be unable to engage in health behaviors because they are busy with work and family commitments and lack the time and mental capacity [46]. Therefore, our cutoff value might be insufficient for specific age groups, and it might vary with individual circumstances. It is also important to individually explore the factors that impede people from implementing health behaviors and to make improvements in their individual circumstances.

Furthermore, the RT analyses revealed there was a group of females who had more interest in health and engaged in more health behaviors than did males, and the proportion of females in the lower- and lowest-health-interest groups was lower than that of males. In Japan, the average life expectancy for females is 87.6 years—longer than that of males at 81.5 years [50]. There is a disparity in life expectancy between males and females. Our findings suggested that females have a greater interest in health and implement more health behaviors as a result; this might be one reason for the disparity in life expectancy between males and females in Japan.

We found a tendency for the percentages of those of lower socioeconomic status to be greater in the lower- and lowest-health-interest groups. Indeed, various factors, including lower socioeconomic status, have been reported to be involved in poorer health behaviors [8,9,10]. Our findings support the findings of these previous reports.

There were some limitations to our study. The first limitation was sampling bias due to the nature of the internet survey. Compared with the participants in the 2022 National Survey of Living Conditions, our participants were younger, included more smokers, and had higher incomes [51,52,53]. Also, our IHS gave overall lower scores for both males and females than those in a previous study targeting people aged in their 30s to 60s [15]. The fact that we included younger people than in this previous study might be one reason for our lower IHS scores. As our findings also suggested that the cutoff value for a lower-health-interest group was similar to the mean IHS value, and IHS and its cutoffs could vary among study participants, it might be also acceptable to use the mean IHS value to classify a lower-health-interest group. This was a limitation, but it could also be an advantage when the IHS score is used in different populations. Secondly, there was a problem with external validity. As we targeted Japanese people, and the IHS was developed only for Japanese, there are limits to its use outside Japan. To use our results overseas in the future, in addition to creating a foreign language version, we will need to compare the cutoffs among countries. Lastly, we have to mention that a reciprocal relationship might exist between IHS and health behavior, whereby higher levels of health behavior could also reinforce an individual’s interest in health.

## 5. Conclusions

Here, we defined the cutoff values of the IHS as less than 32 for a lower-health-interest group and less than 25 for the lowest-health-interest group. If these cutoff values were used to clarify the characteristics of low-health-interest people, who are a high-risk population, policymakers and healthcare professionals might be able to identify them more efficiently and approach them with strategies.

## Figures and Tables

**Table 1:** Baseline participant characteristics.

	N (%)	IHS Score, Mean (SD)	*p* Value
ALL	22,263 (100%)	32.1 (5.6)	
SEX			
male	11,045 (49.6%)	31.6 (5.6)	<0.001 *
female	11,218 (50.4%)	32.7 (5.6)	
AGE (years)			
20–24	3418 (15.4%)	31.6 (5.6)	<0.001 ^†^
25–34	4509 (20.3%)	31.5 (5.6)	
35–44	4728 (21.2%)	31.6 (5.6)	
45–54	5215 (23.4%)	32.2 (5.7)	
55–64	4393 (19.7%)	33.7 (5.4)	
EDUCATION			
junior high school	192 (0.9%)	29.5 (6.1)	<0.001 ^†^
high school	4912 (22.1%)	31.3 (5.6)	
vocational school/college	5003 (22.5%)	32.2 (5.7)	
university or higher	12,011 (54.0%)	32.5 (5.6)	
Others	145 (0.7%)	30.4 (6.1)	
OCCUPATIONAL STATUS			
full-time employee	10,640 (47.8%)	32.0 (5.5)	0.001 ^†^
part-time employee	4518 (20.3%)	32.2 (5.8)	
self-employed/employer	1768 (7.9%)	32.2 (5.4)	
non-worker	3779 (17.0%)	32.5 (5.9)	
student	1558 (7.0%)	31.9 (5.5)	
HOUSEHOLD INCOME			
<3 million yen	3409 (15.3%)	31.5 (6.0)	<0.001 ^†^
3 to less than 5 million yen	4252 (19.1%)	31.7 (5.5)	
5 to less than 7 million yen	3637 (16.3%)	32.1 (5.4)	
7 to less than 10 million yen	3843 (17.3%)	32.9 (5.5)	
≥10 million yen	2562 (11.5%)	33.6 (5.8)	
unknown/disclosed	4560 (20.5%)	31.7 (5.5)	
MARITAL STATUS			
married	11,676 (52.4%)	32.7 (5.4)	<0.001 ^†^
never married	9114 (40.9%)	31.4 (5.8)	
widowed	183 (0.8%)	33.2 (5.7)	
divorced	1290 (5.8%)	31.9 (6.0)	

IHS scores are reported as means ± standard deviation. Discrete variables are expressed as numerals (percentages). The subjects were allocated to groups according to their backgrounds, and the statistical significance of IHS score differences between groups was tested by using Welch’s *t*-test for normally distributed data, the Wilcoxon rank sum test for non-normally distributed data, and one-way analysis of variance for three groups or more. A two-tailed *p* value of <0.05 was considered to indicate statistical significance. Abbreviations: IHS, the Interest in Health Scale; SD, standard deviation. * Welch’s *t*-test; ^†^ one-way analysis of variance.

**Table 2:** ROC curve analyses for each health behavior and the IHS.

	Cut-Off Value	Sensitivity	Specificity	AUC
Currently non-smoker	32.5	0.65	0.49	0.60
No harmful alcohol use	31.5	0.53	0.57	0.55
Sleep for at least 6 h	31.5	0.53	0.59	0.57
Not obese	30.5	0.47	0.64	0.58
Eat breakfast	31.5	0.62	0.60	0.63
Have a nutritionally balanced diet	31.5	0.76	0.64	0.76
Maintain a regular routine	31.5	0.72	0.62	0.73
Brush teeth	32.5	0.79	0.48	0.67
Have received a medical checkup within the past year	31.5	0.53	0.61	0.59
Have received a dental checkup within the past year	32.5	0.61	0.55	0.61

The ROC curves were described with each of the 10 individual health behavior scores (0 or 1 point) as dependent variables and the IHS as an explanatory variable. We calculated the AUC and determined the cutoff value by using the Youden-Index. Abbreviations: AUC, area under the curve; IHS, Interest in Health Scale; ROC, receiver operating characteristic.

**Table 3:** Participant characteristics classified by the cut-offs determined in this study.

	Lowest-Health-Interest Group (IHS < 25)	Lower-Health-Interest Group (25 ≤ IHS < 32)	Normal- or High-Health-Interest Group (IHS ≥ 32)
ALL	1780 (100%)	8181 (100%)	12,302 (100%)
SEX			
male	1044 (58.7%)	4405 (53.8%)	5596 (45.5%)
female	736 (41.3%)	3776 (46.2%)	6706 (54.5%)
AGE (years)			
20–24	323 (18.1%)	1366 (16.7%)	1729 (14.1%)
25–34	426 (23.9%)	1825 (22.3%)	2258 (18.4%)
35–44	452 (25.4%)	1831 (22.4%)	2445 (19.9%)
45–54	406 (22.8%)	1913 (23.4%)	2896 (23.5%)
55–64	173 (9.7%)	1246 (15.2%)	2974 (24.2%)
EDUCATION			
junior high school	47 (2.6%)	71 (0.9%)	74 (0.6%)
high school	490 (27.5%)	2014 (24.6%)	2408 (19.6%)
vocational school/college	388 (21.8%)	1776 (21.7%)	2839 (23.1%)
university or higher	827 (46.5%)	4260 (52.1%)	6924 (56.3%)
Others	28 (1.6%)	60 (0.7%)	57 (0.5%)
OCUPATIONAL STATUS			
full-time employee	827 (46.5%)	4079 (49.9%)	5734 (46.6%)
part-time employee	385 (21.6%)	1590 (19.4%)	2543 (20.7%)
self-employed/employer	120 (6.7%)	667 (8.2%)	981 (8.0%)
non-worker	325 (18.3%)	1248 (15.3%)	2206 (17.9%)
student	123 (6.9%)	597 (7.3%)	838 (6.8%)
HOUSEHOLD INCOME			
<3 million yen	376 (21.1%)	1324 (16.2%)	1709 (13.9%)
3 to less than 5 million yen	374 (21.0%)	1655 (20.2%)	2223 (18.1%)
5 to less than 7 million yen	273 (15.3%)	1339 (16.4%)	2025 (16.5%)
7 to less than 10 million yen	220 (12.4%)	1283 (15.7%)	2340 (19.0%)
≥10 million yen	128 (7.2%)	787 (9.6%)	1647 (13.4%)
unknown/disclosed	409 (23.0%)	1793 (21.9%)	2358 (19.2%)
MARITAL STATUS			
married	662 (37.2%)	4029 (49.2%)	6985 (56.8%)
never married	991 (55.7%)	3600 (44.0%)	4523 (36.8%)
widowed	11 (0.6%)	54 (0.7%)	118 (1.0%)
divorced	116 (6.5%)	498 (6.1%)	676 (5.5%)

Participants were classified into three groups by using the cutoff values that we obtained, namely: (i) normal- or high-health-interest group; (ii) lower-health-interest group; and (iii) lowest-health-interest group. Abbreviation: IHS, Interest in Health Scale.

## Data Availability

The raw data supporting the conclusions of this article will be made available by the authors on request.

## Appendix A

**Table A1:** Question content and answer method used for the Interest in Health Scale.

Questions
HEALTH CONSCIOUSNESS
(1) I’m very self-conscious about my health.
(2) I’m interested in information about my health.
(3) I pay attention to changes in my health condition.
(4) I am more health conscious than people around me.
HEALTH MOTIVATION
(5) I am willing to spend some extra money for my health.
(6) I do everything I can to stay healthy.
(7) We should spend some extra time for health.
(8) I want to put health first in my living.
HEALTH VALUE
(9) Work and income are more important than health.
(10) I worry about my health only when I get sick.
(11) Hobbies and leisure activities are more important than health.
(12) Rather than prevent illness, I just want to be cured when I get sick.

The response format was a 4-point Likert scale, with 4 points for “Agree”, 3 for “Somewhat agree”, 2 for “Somewhat disagree”, and 1 for “Disagree”. The total possible scores therefore ranged from 12 to 48. These questions were developed in Japanese and validated by a study in Japan [13]. This English version questionnaire was reproduced with minor modification to show the contents in English with permission from Wakabayashi, et al., Association between Health Indifference and Problem Drinking using a Nationwide Internet Survey, published by The Japanese Society for Hygiene, 2023 [14].

**Table A2:** Question content, answer method, and definition for each health behavior.

Items	Questionnaire Content, Answer Method, and Definition
(1) Currently non-smoker	“Do you currently smoke? (Please answer for the past 30 days). 1: Almost every day; 2: Sometimes; 3: I used to smoke, but not now (stopped); 4: I never smoke”. Those who answered “3 or 4” to the question were considered “currently non-smokers” (1 point).
(2) No harmful alcohol use	Participants were questioned about their alcohol use by using the Japanese translated version of the Alcohol Use Disorders Identification Test (AUDIT) score [31]. Those whose AUDIT scores were less than 8 points [32] were considered to have “no harmful alcohol use” (1 point).
(3) Sleep for at least 6 h	“During the past month, how much time did you spend sleeping (on average) per day? 1: 0 h; 2: less than 30 min; 3: about 30 min; 4: 1 h; 5: 2 h; 6: 3 h; 7: 4–5 h; 8: 6–7 h; 9: 8–9 h; 10: 10–11 h’ 11: 12 h or more; 12: I don’t know”. Those who answered either “8, 9, 10, or 11” to this question were considered to “sleep for at least 6 h” (1 point).
(4) Not obese	We asked the participants their current body weight and height. Then we calculated their body mass index (BMI = body weight [kg]/height [m]^2^). Those whose BMIs were less than 25.0 kg/m^2^ were considered “not obese” (1 point).
(5) Eat breakfast	“Have you eaten breakfast in the past month? 1: Always; 2: Sometimes; 3: Rarely; 4: Never. Those who answered either “1 or 2” to this question were considered to habitually “eat breakfast” (1 point).
(6) Have a nutritionally balanced diet	“Have you eaten a nutritionally balanced diet in the past month? 1: Always; 2: Sometimes; 3: Rarely; 4: Never”. Those who answered either “1 or 2” to this question were considered to habitually eat a “nutritionally balanced diet” (1 point).
(7) Maintain a regular routine	“Have you tried to maintain a regular routine in the past month? 1: Always; 2: Sometimes; 3: Rarely; 4: Never”. Those who answered either “1 or 2” to this question were considered to “maintain a regular routine” (1 point).
(8) Brush teeth	“How often have you brushed your teeth in the past month? 1: Never; 2: Once a month; 3: 2 or 3 times a month; 4: Once a week; 5: 2 or 3 times a week; 6: 4 or 5 times a week; 7: Almost every day (6 or 7 times a week)”. Those who answered “7” to this question were considered to habitually “brush [their] teeth” (1 point).
(9) Have received a medical checkup within the past year	“Did you undergo any health checkups (medical examinations, health checkups, or comprehensive medical checkups) in the past year? Please do not include cancer-only examinations, maternal checkups, dental checkups, and clinical visits with specific symptoms in your answer. Yes, or No”. Those who answered “Yes” to the question were considered to “have received a medical checkup within the past year” (1 point).
(10) Have received a dental checkup within the past year	“Did you visit a dental clinic for dental checkups or just for cleaning in the past year? Please do not include visits for treatment purposes such as cavities in your answer. Yes or No”. Those who answered “Yes” to this question were considered to “have a dental checkup within the past year” (1 point).

We asked the above 10 questions about health behaviors. Each answer complying with the required behavior was given one point, and the total score was calculated as the health behavior score (score range, 0 to 10).

## References

[1] Rose G. (2001). Sick individuals and sick populations. Int. J. Epidemiol..

[2] Frohlich K.L., Potvin L. (2008). Transcending the known in public health practice: The inequality paradox: The population approach and vulnerable populations. Am. J. Public Health.

[3] Fukuda Y. (2008). Does the population approach increase health inequality? Vulnerable population approach as an alternative strategy. Jpn. J. Hyg..

[4] Roininen K., Tuorila H., Zandstra E.H., de Graaf C., Vehkalahti K., Stubenitsky K., Mela D.J. (2001). Differences in health and taste attitudes and reported behaviour among Finnish, Dutch and British consumers: A cross-national validation of the Health and Taste Attitude Scales (HTAS). Appetite.

[5] Monden S. (2002). Consciousness, knowledge and behavior on life-style related diseases of students. Jpn. J. Public Health.

[6] Koyano W., Ueno M., Imaeda M. (2006). Latent factors generating health behavior and health consciousness. Jpn. J. Public Health.

[7] Fukuda Y., Yamada T., Kumi S., Ozawa C., Ishikawa H. (2024). A discussion about identification and definition of and approach to health-uninterest population. Jpn. J. Health Educ. Promot.

[8] Short S.E., Mollborn S. (2015). Social Determinants and Health Behaviors: Conceptual Frames and Empirical Advances. Curr. Opin. Psychol..

[9] Rosenstock I.M. (1974). The Health Belief Model and Preventive Health Behavior. Health Educ. Monogr..

[10] Champion V.L., Skinner C.S., Glanz K., Rimer B.K., Viswanath K. (2008). The health belief model. Health Behavior and Health Education: Theory, Research, and Practice.

[11] Fukuda Y. (2017). Behavioral economics and health inequalities in population strategies: Introduction of “PROGRESS-Plus” and “CAN” frameworks in Japan. Jpn. J. Health Educ. Promot..

[12] Sugimoto K., Fukuda Y. (2022). Typology of population approaches: Perspectives on the population indifferent to health and health inequalities. Jpn. J. Public Health.

[13] Prochaska J.O., Velicer W.F. (1997). The transtheoretical model of health behavior change. Am. J. Health Promot..

[14] Adams J., White M. (2003). Are activity promotion interventions based on the transtheoretical model effective? A critical review. Br. J. Sports. Med..

[15] Ozawa C., Ishikawa H., Kato M., Fukuda Y. (2021). Development of the Interest in Health Scale to understand the “population indifferent to health”. Jpn. J. Health Educ. Promot..

[16] Wakabayashi M., Ishikawa H., Fukuda Y., Iso H., Tabuchi T. (2023). Association between health indifference and problem drinking using a nationwide internet survey. Environ. Health Prev. Med..

[17] Breiman L., Friedman J., Olshen R.A., Stone C.J. (1984). Classification and Regression Trees.

[18] Lewis R.J. An introduction to Classification and Regression Tree (CART) analysis. Proceedings of the 2000 Annual Meeting of the Society for Academic Emergency Medicine.

[19] Krzywinski M., Altman N. (2017). Classification and regression trees. Nat. Methods.

[20] Marshall R.J. (2001). The use of classification and regression trees in clinical epidemiology. J. Clin. Epidemiol..

[21] Taylor W., Gladman D., Helliwell P., Marchesoni A., Mease P., Mielants H., CASPAR Study Group (2006). Classification criteria for psoriatic arthritis: Development of new criteria from a large international study. Arthritis. Rheum..

[22] D’Alisa S., Miscio G., Baudo S., Simone A., Tesio L., Mauro A. (2006). Depression is the main determinant of quality of life in multiple sclerosis: A classification-regression (CART) study. Disabil. Rehabil..

[23] Fonarow G.C., Adams K.F., Abraham W.T., Yancy C.W., Boscardin W.J., ADHERE Scientific Advisory Committee, Study Group, and Investigators (2005). Risk stratification for in-hospital mortality in acutely decompensated heart failure: Classification and regression tree analysis. JAMA.

[24] Wiegel M., Meston C., Rosen R. (2005). The female sexual function index (FSFI): Cross-validation and development of clinical cutoff scores. J. Sex. Marital Ther..

[25] Möckel M., Müller R., Vollert J., Müller C., Danne O., Gareis R., Störk T., Dietz R., Koenig W. (2007). Lipoprotein-associated phospholipase A2 for early risk stratification in patients with suspected acute coronary syndrome: A multi-marker approach: The North Wuerttemberg and Berlin Infarction Study-II (NOBIS-II). Clin. Res. Cardiol..

[26] Tabuchi T., Shinozaki T., Kunugita N., Nakamura M., Tsuji I. (2019). Study Profile: The Japan “Society and New Tobacco” Internet Survey (JASTIS): A Longitudinal Internet Cohort Study of Heat-Not-Burn Tobacco Products, Electronic Cigarettes, and Conventional Tobacco Products in Japan. J. Epidemiol..

[27] Rakuten Insight Inc What is Rakuten Insight?. https://insight.rakuten.co.jp/beginner/.

[28] JACSIS Study The Japan COVID-19 and Society Internet Survey. https://jacsis-study.jp/.

[29] Tokyo Metropolitan Government Public Opinion Poll on Health as of November 2021. https://www.metro.tokyo.lg.jp/tosei/hodohappyo/press/2021/11/18/documents/01.pdf.

[30] Breslow L., Enstrom J.E. (1980). Persistence of health habits and their relationship to mortality. Prev. Med..

[31] Yatabe H., Sugimori H., Suka M., Iida Y., Nakamura T., Yoshida K. (2001). Development of the new assessment tools for lifestyles: Japanese Health Practice Index (JHPI). Jpn. J. MHTS.

[32] The Japanese Ministry of Health, Labour and Welfare The National Health and Nutrition Survey as of 2022. https://www.mhlw.go.jp/toukei/chousahyo/20-21/dl/koku2022ke.pdf.

[33] Ministry of Health, Labour and Welfare Health Japan 21 (the Third Term). https://www.mhlw.go.jp/content/001102474.pdf.

[34] Komatsu T., Yoshimoto H. (2001). The Alcohol Use Disorders Identification Test Guidelines for Use in Primary Care.

[35] Conigrave K.M., Hall W.D., Saunders J.B. (1995). The AUDIT questionnaire: Choosing a cut-off score. Alcohol Use Disorder Identification Test. Addiction.

[36] Ministry of Health, Labour and Welfare Sleep Guide for Health Promotion 2023 (Plan). https://www.mhlw.go.jp/content/10904750/001181265.pdf.

[37] Yamada Y., Yoshida T., Nakagata T., Nanri H., Miyachi M. (2021). Letter to the Editor: Age, Sex, and Regional Differences in the Effect of COVID-19 Pandemic on Objective Physical Activity in Japan: A 2-Year Nationwide Longitudinal Study. J. Nutr. Health Aging.

[38] Tison G.H., Avram R., Kuhar P., Abreau S., Marcus G.M., Pletcher M.J., Olgin J.E. (2020). Worldwide Effect of COVID-19 on Physical Activity: A Descriptive Study. Ann. Intern. Med..

[39] Ishibashi S., Taniguchi M. (2022). Workstyle change effects on physical activity and health consciousness in Japan: Results from COVID-19 lifestyle activity survey. Transp. Res. Interdiscip. Perspect..

[40] Therneau T.M. Package ‘Rpart’. https://cran.r-project.org/web/packages/rpart/rpart.pdf.

[41] Therneau T.M., Atkinson E.J. An Introduction to Recursive Partitioning Using the Rpart Routines. https://cran.r-project.org/web/packages/rpart/vignettes/longintro.pdf.

[42] Prochaska J.O., DiClemente C.C. (1983). Stages and processes of self-change of smoking: Toward an integrative model of change. J. Consult. Clin. Psychol..

[43] Benach J., Malmusi D., Yasui Y., Martínez J.M. (2013). A new typology of policies to tackle health inequalities and scenarios of impact based on Rose’s population approach. J. Epidemiol. Community Health.

[44] Williams J., Allen L., Wickramasinghe K., Mikkelsen B., Roberts N., Townsend N. (2018). A systematic review of associations between non-communicable diseases and socioeconomic status within low- and lower-middle-income countries. J. Glob. Health.

[45] Bradley A.P. (1997). The use of the area under the ROC curve in the evaluation of machine learning algorithms. Pattern Recognit..

[46] Ministry of Health, Labour and Welfare The National Health and Nutrition Survey as of 2019. https://www.mhlw.go.jp/content/10900000/000687163.pdf.

[47] Honjo K., Iso H., Inoue M., Tsugane S., JPHC Study Group (2010). Smoking cessation: Predictive factors among middle-aged Japanese. Nicotine Tob. Res..

[48] Japan Dental Association Consumer Awareness Survey Regarding Dental Care as of 20 October 2022. https://www.jda.or.jp/jda/release/cimg/2022/DentalMedicalAwarenessSurvey_R4.pdf.

[49] Lasko T.A., Bhagwat J.G., Zou K.H., Ohno-Machado L. (2005). The use of receiver operating characteristic curves in biomedical informatics. J. Biomed. Inform..

[50] Ministry of Health, Labour and Welfare Life Table as of 2021 (International Comparison of Average Life Expectancy). https://www.mhlw.go.jp/toukei/saikin/hw/life/life21/dl/life21-04.pdf.

[51] Statistics Bureau of Japan Overview of Population Projections as of October 2022. https://www.stat.go.jp/data/jinsui/2022np/pdf/2022gaiyou.pdf.

[52] Ministry of Health, Labour and Welfare The National Health and Nutrition Survey as of 2022 (Income Status of Various Households). https://www.mhlw.go.jp/toukei/saikin/hw/k-tyosa/k-tyosa22/dl/03.pdf.

[53] Ministry of Health, Labour and Welfare The National Health and Nutrition Survey as of 2022 (Health Status of Household Members). https://www.mhlw.go.jp/toukei/saikin/hw/k-tyosa/k-tyosa22/dl/04.pdf.

